# Dynamic Variation in the Semiconductive Tendency of the Passive Film on Duplex Stainless Steel in Corrosion Environments

**DOI:** 10.3390/ma17235963

**Published:** 2024-12-05

**Authors:** Seung-Heon Choi, Young-Ran Yoo, Young-Sik Kim

**Affiliations:** 1 Department of Materials Science and Engineering, Andong National University, 1375 Gyeongdong-ro, Andong 36729, Gyeongbuk, Republic of Korea; csh140541@pyunji.andong.ac.kr; 2 Materials Research Centre for Energy and Clean Technology, Andong National University, 1375 Gyeongdong-ro, Andong 36729, Gyeongbuk, Republic of Korea; yryoo@anu.ac.kr

**Keywords:** stainless steel, passive film, electrochemical properties, semiconductive tendency

## Abstract

Duplex stainless steels, known for their excellent corrosion resistance, are employed in a variety of chloride solutions—acidic, neutral, and alkaline—due to a stable passive film that forms on their surfaces. This study involved polarization tests, EIS (Electrochemical Impedance Spectroscopy) measurements, Mott–Schottky plots, and XPS (X-Ray Photoelectron Spectroscopy) analyses in both static and dynamic conditions across acidic (1NaCl + 0.1N HCl, pH 1.0), neutral (1N NaCl, pH 6.7), and alkaline (1N NaCl + 0.1N NaOH, pH 13.2) chloride solutions to confirm that duplex stainless steels exhibit similar passivation behavior (0.79 μA/cm^2^ > i_p_ > 0.2 μA/cm^2^ and 590 kΩ·cm^2^ < R_p_ < 651 kΩ·cm^2^). Regardless of the pH of the solution, p-type and n-type semiconductive properties were observed, but the balance of the semiconductive tendencies was different. Comparing passive films formed under dynamic conditions, through real-time HCl injection into a neutral chloride solution, with those formed under static conditions, revealed that both conditions yield similar structural and property characteristics in the films, as well as comparable electrochemical behaviors. These findings suggest that the passive film on the stainless steel surface adjusts to the environment and can be spontaneously repassivated in response to environmental changes.

## 1. Introduction

Stainless steel, an alloy primarily containing iron and at least 12% chromium, is extensively utilized across various industrial sectors for its outstanding corrosion resistance and mechanical properties. The formation of a passive film on the metal surface is essential to its corrosion protection, effectively shielding the metal from environmental exposure [1]. This passive film predominantly consists of oxides or hydroxides of iron and chromium [2,3,4].

There are two main theories of passive films. The first is the “oxide film theory”, which posits that passive films are diffusive barrier layers formed from reaction products such as metal oxides [5]. The second, known as the “adsorption theory”, suggests that oxygen absorbed on the metal surface displaces water molecules and inhibits the hydration of metal ions, thereby reducing the dissolution reaction [6]. Further theories, such as the “Electron Configuration-Induced Adsorption Passivation” and the “Ionic Space Charge-Induced Passivation”, offer additional perspectives on the protective mechanisms of passive films [7,8,9].

Early studies of passive films primarily employed XPS to analyze their thickness and composition. These studies revealed that passive films on Fe-Cr and Fe-Cr-Ni alloys predominantly consist of Cr and Fe oxides, with Mo^6+^ states observed in the outer layer when Mo is added [10,11,12,13,14]. However, variations in passive film composition and properties, along with the limitations of ex situ techniques, have led to divergent views on passive film behavior. The discovery of electric fields within passive films has established photocurrent measurement and Mott–Schottky analysis as critical methods for studying their semiconducting properties. Two key models dominate the understanding of semiconductive properties in stainless steel passive films. The “point defect model” explains the growth and destruction of films based on oxygen and metal vacancies, providing insight into corrosion resistance [15,16,17,18,19,20,21,22]. The “bipolar fixed charge-induced passivity” model highlights the roles of Cr and Mo in influencing ion selectivities within the film [23,24,25,26,27,28,29,30,31,32]. Despite these advances, experimental evidence on semiconductor properties remains limited.

Studies utilizing Mott–Schottky analysis to investigate the semiconductive properties of stainless steel passive films have revealed a variety of behaviors depending on alloy composition and environmental conditions. For instance, passive films on AISI 321 alloy in 0.5 M H_2_SO_4_ showed exclusively n-type behavior [33], while pure Fe in borate buffer (pH 8.5) exhibited diminishing n-type properties with increasing temperature [34]. Fe-20Cr alloys with added Ni and Mo displayed both p-type and n-type slopes, though primarily characterized by n-type behavior [16]. Additional studies demonstrated that Si content in Fe-20Cr alloys amplified both p-type and n-type behaviors, with n-type dominating [35]. AISI 304 alloys showed varied semiconductive properties depending on the solution pH, exhibiting both p-type and n-type behavior in acidic environments [36,37]. Research on AISI 316LN alloys highlighted that nitrogen content enhances both p-type and n-type tendencies, with significant amplification at neutral pH [38]. Overall, these findings indicate that passive films on stainless steels can exhibit either n-type or p-type properties, or a combination of both, influenced by alloy composition and environmental factors.

From a semiconductor perspective, passive films can be interpreted as n-type semiconductors in certain environments, or they may exhibit both p-type and n-type semiconductive properties. The interpretation of the semiconductive properties of the passive film varies depending on the environment. Furthermore, the magnitude of the Mott–Schottky slope also varies with the environment, exhibiting either smaller or larger slopes. Some studies have extended the “point defect model” to interpret these slopes. Previous research has broadened our understanding of the semiconductive properties of passive films through the application of the “bipolar model”, which highlights that the primary factor influencing passivation behavior is the balance between p-type and n-type semiconductive tendencies [39]. Our study on SR-50A stainless steel under static conditions in acidic and alkaline chloride environments demonstrated that adjusting the pH to achieve a balanced ratio of p-type and n-type semiconductive properties is critical for enhancing the stability and corrosion resistance of passive films. Specifically, it revealed that in extreme pH conditions, the dominance of either p-type or n-type behavior leads to destabilization of the passive film. Conversely, a balanced semiconductive property significantly improves the robustness of the passive film, providing resistance to environmental changes. These findings laid the groundwork for the current investigation.

Most of the existing research has examined the semiconductive properties of passive films in acidic, neutral, and alkaline solutions under static conditions (in this work, static condition means that the concentration of a solution remains constant with time). However, actual corrosion environments may become locally acidic or alkaline and deviate from the initial conditions over time. Under static conditions, the concentration of a solution remains uniform from its initial state, but in dynamic conditions, the concentration can be altered by various external factors. These localized environmental changes promote localized corrosion, and conditions that are initially only mildly corrosive can gradually become more corrosive over time.

Building upon our previous findings, this study focuses on duplex stainless steel and investigates the semiconductive properties of passive films under pH-adjusted acidic, neutral, and alkaline conditions. Using anodic polarization tests, regions exhibiting similar passivation behavior across these environments were identified, and the influence of p-type and n-type semiconductive tendencies was analyzed. Furthermore, this study examines electrochemical properties, semiconductive behavior, and XPS data under both static and dynamic conditions to provide a comprehensive understanding of passive film changes and stability in realistic corrosion scenarios.

## 2. Materials and Methods

### 2.1. Experimental Alloy

**Figure 1:**
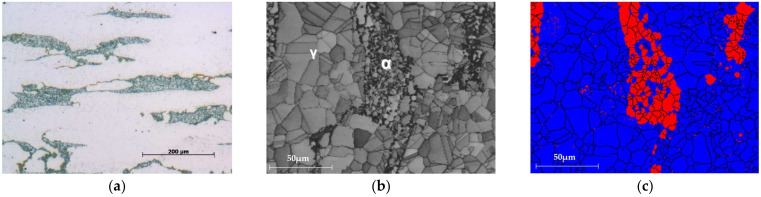
Optical microstructure and EBSD analysis of duplex stainless steel: (**a**) OM (×200), (**b**) band contrast (×700), and (**c**) phase color (×700).

To investigate the semiconductive properties of the passive film on duplex stainless steel under different corrosion environments, a duplex stainless steel with a PREN_30_ value of 46.1 was used, demonstrating excellent corrosion resistance. This high-nickel duplex stainless steel contains 10.7% nickel (Ni). The chemical composition of the alloy used in the experiment is presented in Table 1. Figure 1 shows the microstructure of the duplex stainless steel. In Figure 1a, α-ferrite and γ-austenite phases are observed through optical microscopy (AXIOTECH 100 HD, ZEISS, Oberkochen, Germany), and Figure 1b displays the clear boundary between the two phases in a band contrast image captured via EBSD (Oxford Instruments, Bognor Regis, UK). In Figure 1b, the α region corresponds to the ferrite phase, and the γ region corresponds to the austenite phase. Figure 1c, a phase color image captured via EBSD (Oxford Instruments, Bognor Regis, UK), visually confirms that the alloy consists of 85.4% austenite and 14.6% ferrite phases approximately.

### 2.2. Polarization Test

The polarization experiments were conducted to evaluate the corrosion and passivation characteristics. The specimens were cut into 1.5 cm × 1.5 cm sections, spot-welded with copper wire for electrical contact, and sealed with epoxy resin. The specimen surfaces were sequentially polished using SiC paper from #80 to #2000 grit, with an exposed area restricted to 1 cm^2^ and the remaining portion insulated with epoxy resin. The experiments were conducted using a potentiostat (ZIVE MP1, Won A Tech, Daejeon, Republic of Korea), with a platinum (Pt) counter electrode and a saturated calomel electrode (SCE) as the reference. The test solution was prepared by adding 0.1N HCl and 0.1N NaOH, respectively, to a neutral saline solution (1N NaCl) to create acidic and alkaline environments, and all the solutions were deaerated. The experiments proceeded at a scan rate of 0.33 mV/s, and the corrosion current density was calculated using the Tafel extrapolation method within a range of ±100 mV (SCE) from the corrosion potential [40].

### 2.3. Potentiostatic EIS Test

**Figure 2:**
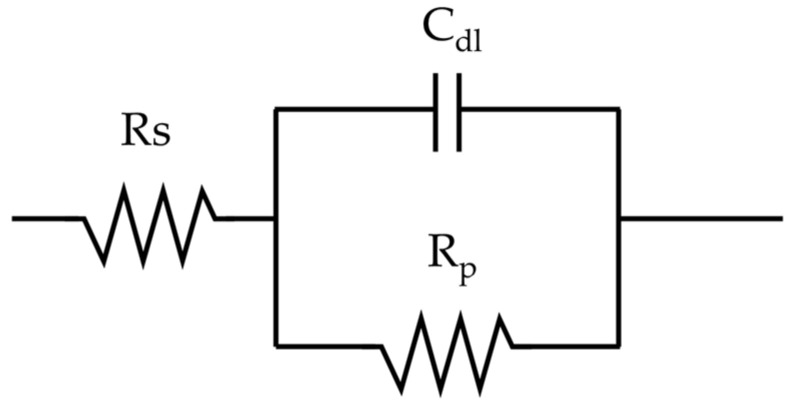
Randles model [40].

The resistance of the passive film was evaluated through potentiostatic Electrochemical Impedance Spectroscopy (EIS) testing. The specimens and experimental solutions were prepared in the same way as for the anodic polarization experiments. Using a constant potential apparatus (Interface 1000, Gamry Instruments, Warminster, PA, USA), the passive film was formed for 30 min at −100 mV (SCE), the passivation zone potential was obtained from the anodic polarization curve, and then the impedance was measured at the same potential. The frequency range was set from 10 kHz to 0.01 Hz (10 points per decade) with an AC voltage amplitude of 10 mV and the polarization resistance (R_p_), the solution resistance (R_s_), and the capacitance (C_dl_) of the passivation film were calculated using the Randles model [39,40] (Figure 2).

### 2.4. Mott–Schottky Analysis

To analyze the semiconductor properties of the passivation films, the specimens and experimental solutions were prepared in the same way as for the electrochemical experiments. For passive film formation, a potential of −100 mV (SCE) was maintained for 30 min using a potentiostat (Interface 1000, Gamry Instruments, Warminster, PA, USA), followed by a Mott–Schottky analysis. The experiments were performed with the AC amplitude fixed at 10 mV (peak to peak) and the frequency set at 1580 Hz. The measured potential ranged from +1 V (SCE) to −1 V (SCE) in acidic environments and +1 V (SCE) to −1.5 V (SCE) in neutral and alkaline environments, and the capacitance was measured at a rate of 50 mV/s. To calculate the donor (N_D_) and acceptor (N_A_) densities from the Mott–Schottky plot, linear fitting was performed on the linear portions of the p-type and n-type semiconductor regions. The average slope obtained from this fitting was used for the calculations.

### 2.5. XPS

The specimens for passive film analysis were polished to a mirror finish using 1 μm diamond paste. To form the stable passive film, a potentiostat (ZIVE MP1, Won A Tech, Republic of Korea) was used to form the passive film at −100 mV (SCE) in deaerated neutral, acidic, and alkaline chloride solutions for 2 h. After formation, the passivated specimens were stored in a nitrogen atmosphere until analysis. X-ray Photoelectron Spectroscopy (XPS) was conducted using a K-alpha instrument (Thermo UK, Altrincham, UK). The elemental spectra were collected with a Al-Kα X-ray source (1486.6 eV, 12 kV, 3 mA). Depth profiling was performed with Ar-sputtering at 500 V and 2 μA, with sputtering time intervals set at 20 s per layer. After the XPS measurements, the chemical states of each element were analyzed using Avantage software (Version 6.8.1.4, Thermo Fisher Scientific, Waltham, MA, USA), calibrated to the C 1s spectrum binding energy (284.6 eV).

### 2.6. Dynamic Electrochemical Test

In order to determine the changes in the properties of the passive films due to environmental changes, we first evaluated the properties of the passivated films in a neutral chloride solution (1N NaCl, pH 6.7) in the static condition and then performed electrochemical evaluations by injecting 0.1 N HCl into the neutral chloride solution in real time (in this work, this implies the dynamic condition). In addition, XPS analyses were performed on the passive films by the static and dynamic conditions.

## 3. Results and Discussion

### 3.1. Electrochemical Properties and Semiconductive Tendenies in Chloride Solutions Under Static Conditions

**Figure 3:**
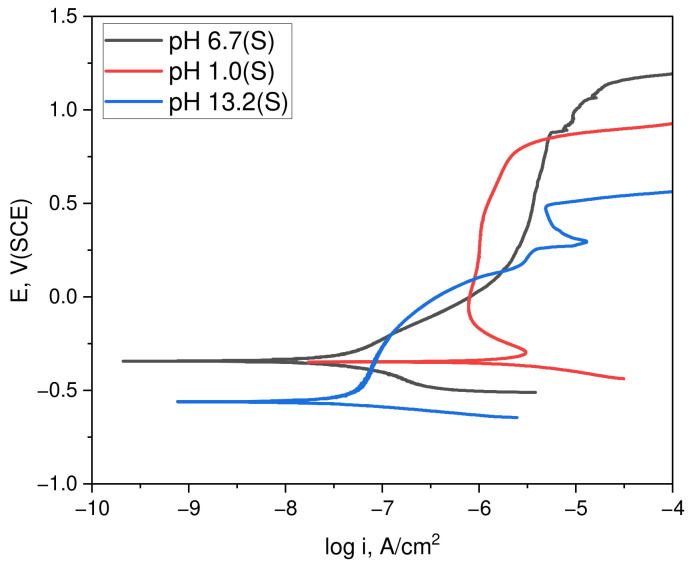
Effect of corrosive environments on the polarization curves of duplex stainless steel.

Figure 3 details the polarization behavior of duplex stainless steel in acidic (1N NaCl + 0.1N HCl, pH 1.0) and alkaline chloride (1N NaCl + 0.1N NaOH, pH 13.2) environments, founded on a static neutral chloride (1N NaCl, pH 6.7) solution deaerated at 25 °C. The experiments compared the polarization behavior through the corrosion potential (E_R_), corrosion current density (i_R_), transpassive potential (E_tr_), and passive current density (i_P_) measured at −100 mV (SCE). The corrosion potential (E_R_) ranged from −0.34 V (SCE) to −0.35 V (SCE) as the pH decreased and reached −0.56 V (SCE) in the alkaline environment. Additionally, the corrosion current density (i_R_) was +0.02 μA/cm^2^ in the neutral environment but increased significantly to +0.03 μA/cm^2^ in the alkaline environment and +1.39 μA/cm^2^ in the acidic environment [20]. For the transpassive potential (E_tr_), values of +1.12 V (SCE) in the neutral environment, +0.83 V (SCE) in the acidic environment, and +0.49 V (SCE) in the alkaline environment were recorded [16]. This indicates the highest transpassive potential in the neutral environment, with a decrease observed in the acidic and alkaline environments.

The passive current density (i_P_) measured at −100 mV (SCE) was +0.35 μA/cm^2^, +0.79 μA/cm^2^, and +0.20 μA/cm^2^ in the neutral, acidic, and alkaline environments, respectively. This suggests that with an increasing pH, either the passive film on duplex stainless steel thickens or a more stable passive film forms in the −100 mV (SCE) environment. Table 2 summarizes the polarization factors for each pH condition

**Figure 4:**
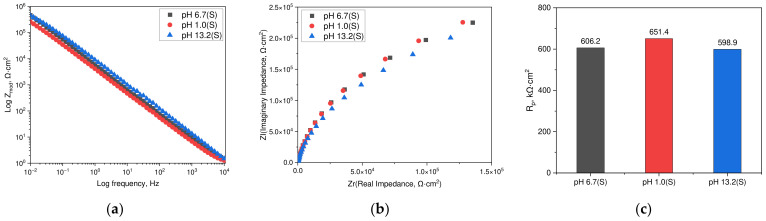
EIS results in the passive film of duplex stainless steel formed at −100 mV (SCE) under various conditions: (**a**) Bode plot, (**b**) Nyquist plot, and (**c**) polarization resistance by CPE model.

Figure 4 shows the results of the EIS measurements on the passive films formed in acidic (1N NaCl + 0.1N HCl, pH 1.0) and alkaline chloride (1N NaCl + 0.1N NaOH, pH 13.2) environments, as well as in a static neutral chloride solution (1N NaCl, pH 6.7) that was deaerated at 25 °C. The passive films were analyzed after 1 h at the passive potential of the polarization curve (−100 mV (SCE)). Figure 4a is a Bode plot, indicating that impedance values decrease at low- and high-frequency regions across all environments. Figure 4b presents a Nyquist plot, illustrating that the size of the semicircles was similar in all environments. The results in Figure 4a,b were analyzed using the Randles model to calculate the polarization resistance values, presented in Figure 4c. These were calculated to be 651.4 kΩ·cm^2^ in neutral chloride, 606.2 kΩ·cm^2^ in acidic environments, and 598.9 kΩ·cm^2^ in alkaline environments.

**Figure 5:**
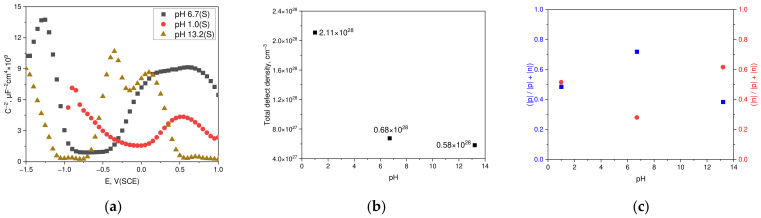
Effect of corrosive environment on the Mott–Schottky behavior of passive film on duplex stainless steel formed at −100 mV (SCE): (**a**) Mott–Schottky plot, (**b**) pH vs. total defect density, and (**c**) pH vs. p-type or n-type semiconductive tendency.

Figure 5 illustrates the Mott–Schottky behavior of the passive films formed by applying a potential of −100 mV (SCE) for 1 h in the acidic and alkaline environments, using a static neutral chloride solution deaerated at 25 °C. The Mott–Schottky plot shows positive slopes indicating n-type semiconductor characteristics and negative slopes indicating p-type semiconductor characteristics

Figure 5a presents the results of the Mott–Schottky analysis for the passive films formed in each environment. For neutral chloride, a negative slope ranges from −1.25 V to −0.95 V (SCE) and a positive slope from −0.35 V to 0 V (SCE), showing strong p-type and n-type behavior, respectively. This trend is consistent with other studies [16,17,18,19,22,34,35,36]. In alkaline chloride solutions, negative slopes occur between −1.5 V and −1.1 V (SCE) and positive slopes between −0.7 V and −0.4 V (SCE), matching those in neutral chloride. This trend aligns with the findings from previous studies [41,42,43,44]. In acidic chloride solutions, a negative slope is observed from −0.85 V to −0.4 V (SCE) and a positive slope from 0.1 V to 0.4 V (SCE), generally indicating weaker p-type and n-type behaviors. This result also corresponds to other studies [16,20,21,33]. The semiconductive properties of the passive film were analyzed using the point defect model, where donor and acceptor concentrations were calculated from the p-type and n-type slopes in each environment. The resulting total defect densities are shown in Figure 5b, demonstrating that the total defect density of the passive film formed at −100 mV (SCE) tends to increase as the pH decreases [20]. Figure 5c illustrates the correlation between p-type and n-type semiconductive tendencies across various pH environments, using the bipolar model [38]. In a neutral chloride environment, the p-type semiconductive tendency predominated over the n-type. Conversely, in the acidic and alkaline chloride environments, a shift occurred, with n-type tendencies surpassing p-type. Notably, in the acidic environment, the p-type and n-type semiconductive tendencies were almost balanced.

Table 3 presents the flat band potential, donor density, acceptor density, and total defect density of the passive film formed on duplex stainless steel in chloride solutions of various pH. The flat band potential (E_fb_) was defined as the potential at which the C^−2^ value becomes zero, determined using the slope of the tangent lines depicting p-type and n-type characteristics in the Mott–Schottky plot. As the pH increased, the flat band potential (E_fb_) generally exhibited a decreasing trend.

An XPS analysis was conducted to investigate the chemical states of the elements in the outer and inner layers of the passive films formed in acidic, neutral, and alkaline chloride environments. For the acidic and neutral chloride environments, etching times of 20 and 80 s were used to differentiate the outer and inner layers; in the alkaline environments, these times were set at 20 and 120 s for the outer and inner layers, respectively.

**Figure 6:**
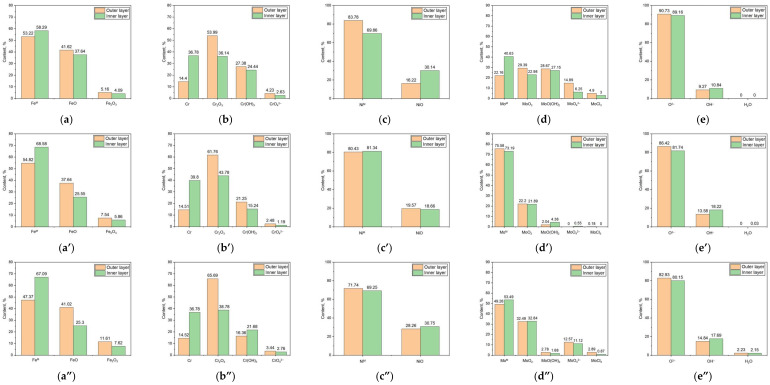
(**a**,**a’**,**a”**) Fe 2p_3/2_, (**b**,**b’**,**b”**) Cr 2p_3/2_, (**c**,**c’**,**c”**) Ni 2p_3/2_, (**d**,**d’**,**d”**) Mo (3d_5/2_ + 3d_3/2_), and (**e**,**e’**,**e”**) O 1s elemental composition of outer and inner layers of passive films under (**a**–**e**) acidic, (**a’**–**e’**) neutral, and (**a”**–**e”**) alkaline chloride solution.

Figure 6 shows the Fe 2p_3/3_, Cr 2p_3/3_, Ni 2p_3/3_, Mo (3p_5/2_ + 3p_3/2_), and O 1s elemental composition of the outer and inner layers of the passive films under different solution environments. Figure 6a,a’,a” display the Fe 2p_3/2_ chemical states of the outer and inner layers of the passive films formed in the acidic, neutral, and alkaline chloride environments. Regardless of the solution in which the passive film was formed, the chemical states of Fe 2p_3/2_ included Fe^M^, FeO, and Fe_2_O_3_. Fe^M^ was predominantly found in the inner layer, while FeO was concentrated mainly in the outer layer, and Fe_2_O_3_ was evenly distributed across both layers. Figure 6b,b’,b” illustrate the Cr 2p_3/2_ chemical states for both the outer and inner layers of the passive films formed in acidic, neutral, and alkaline chloride environments. The chemical states of Cr 2p_3/2_ included Cr^M^, Cr_2_O_3_, Cr(OH)_3_, and CrO_4_^2−^ in all the environments. For Cr^M^, the concentration tended to increase within the inner layers across all the acidic, neutral, and alkaline environments. Cr_2_O_3_ predominantly resided in the outer layer of the passive films regardless of the environment but showed the greatest concentration in the alkaline conditions, followed by the neutral and acidic contexts. Cr(OH)_3_ was predominantly found in the outer layer in the acidic and neutral environments, yet it was concentrated in the inner layer in the alkaline conditions. CrO_4_^2−^ was observed to be concentrated primarily in the outer layer of the passive films under all the conditions. Figure 6c,c’,c” present the Ni 2p_3/2_ chemical states for both the outer and inner layers of the passive films formed in the acidic, neutral, and alkaline chloride environments. The chemical states of Ni 2p_3/2_ comprised Ni^M^ and NiO in all the conditions. Ni^M^ was concentrated in the outer layer in the acidic and alkaline environments, whereas in the neutral environments, it was distributed fairly equally between the outer and inner layers. NiO was found primarily in the inner layer in the acidic and alkaline environments but was evenly distributed in the outer and inner layers in the neutral environments. Figure 6d,d’,d” reveal the Mo (3p_5/2_ + 3p_3/2_) chemical states for the outer and inner layers of the passive films formed in the acidic, neutral, and alkaline chloride environments. The chemical states of Mo (3p_5/2_ + 3p_3/2_) consisted of Mo^M^, MoO_2_, MoO(OH)_2_, MoO_4_^2−^, and MoCl_2_ irrespective of the environment. Mo^M^ primarily increased in concentration toward the inner layer in the acidic and alkaline conditions, while in the neutral environments it was more prevalent in the outer layer. MoO_2_ was distributed evenly between the outer and inner layers in the neutral and alkaline conditions but was concentrated in the outer layer in the acidic settings. MoO(OH)_2_ was mainly detected in the acidic environments, with its concentration diminishing toward the inner layer. MoO_4_^2−^ generally decreased in concentration toward the inner layer in the acidic and alkaline conditions and was scarcely detected in the neutral environments. MoCl_2_ also showed a decreasing concentration toward the inner layers in the acidic and alkaline environments and was only minimally detected in the neutral settings. Figure 6e,e’,e” illustrate the chemical states of O 1s in the outer and inner layers of the passive films formed in the acidic, neutral, and alkaline chloride environments. These chemical states were analyzed as O^2−^, OH^−^, and H_2_O. The concentration of O^2−^ generally decreased from the outer to the inner layer, regardless of the environment, while the concentration of OH^−^ increased. Notably, the concentration of OH^−^ ions in the alkaline solutions was higher than in the other solutions, indicating that passive films formed in alkaline environments are thicker than those in acidic and neutral environments. H_2_O was observed only in traces in the alkaline environment.

**Figure 7:**
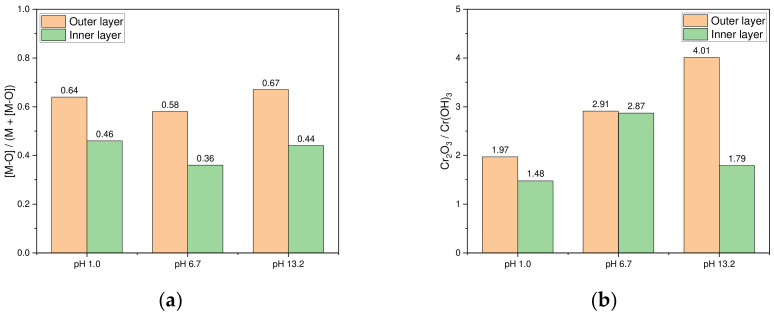
[M-O]/(M + [M-O]) ratio and Cr_2_O_3_/Cr(OH)_3_ ratio for the outer and inner layers of the passive film formed in the acidic, neutral, and alkaline chloride environments ([M-O] means metal-oxide): (**a**) ratio of [M-O]/(M + [M-O]) and (**b**) ratio of Cr_2_O_3_/Cr(OH)_3_.

Figure 7 presents the [M-O]/(M + [M-O]) and Cr_2_O_3_/Cr(OH)_3_ ratios in the outer and inner layers of the passive films across the acidic, neutral, and alkaline chloride environments. Figure 7a shows the decreasing trend of the [M-O]/(M + [M-O]) ratio from the outer layer to the inner layer in each environment. Figure 7b illustrates that in the acidic and alkaline environments, the Cr_2_O_3_/Cr(OH)_3_ ratio is higher in the outer layer, peaking in the alkaline environment. In neutral environments, the Cr_2_O_3_/Cr(OH)_3_ ratio is almost equally distributed between the outer and inner layers. Overall, this ratio is highest in the alkaline environments, followed by the acidic and neutral environments. These findings are consistent with the results reported in previous studies [11,12,13,14,28,30,37].

In general, metals exhibit the highest corrosion resistance in neutral environments while experiencing reduced resistance in acidic or alkaline conditions. This study adjusted the concentrations of acidic and alkaline chloride solutions to form passive films similar to those in neutral chloride solutions; the passive current density at a potential of −100 mV (SCE) was maintained within a range of +0.2 to +0.79 μA/cm^2^, and the polarization resistance ranged from 590 to 651 kΩ·cm^2^, indicating comparable corrosion resistance. Despite varying corrosion strengths in the acidic and alkaline solutions, the corrosion resistance remains excellent. Examining the main influences on passivation behavior, it is noted that the i_p_ and R_p_ values measured are similar across different environments in this work, implying these factors are not crucial for corrosion resistance. Moreover, the Cr_2_O_3_/Cr(OH)_3_ ratio, which is crucial in the corrosion resistance of stainless steel, was found to be lower in acidic environments compared to other conditions. Therefore, it showed no significant effect on passivation behavior, suggesting that this ratio may not be a critical factor. From a semiconductor perspective, the p-type and n-type properties in acidic solutions are slightly weaker than in other environments, yet the balance between these properties is maintained, contributing to effective corrosion resistance. Thus, it is concluded that the key factor influencing passive behavior in acidic environments is the equilibrium between the semiconductor properties—specifically, the balance of p-type and n-type characteristics, which facilitates the stability and effectiveness of passive films against corrosion.

On the other hand, the passivation properties in neutral and alkaline environments display strong p-type and n-type characteristics without achieving balance. Despite this, effective passivation is still observed, indicating that a perfect balance of semiconductor properties is not necessary for maintaining passivation behavior in these environments. Nevertheless, good passivation behavior was maintained in the neutral and alkaline environments, suggesting that the balance of semiconductive properties does not significantly affect passivation behavior in neutral and alkaline environments. Enhanced corrosion resistance in neutral solutions results from the less corrosive environment, whereas, in alkaline conditions, superior corrosion resistance is primarily attributed to the higher stability of oxides. The XPS analysis has shown that oxide thickness in alkaline environments exceeds that in acidic conditions. However, the precise factors influencing the corrosion resistance of passive films in these solutions remain uncertain, necessitating further research for clarification.

### 3.2. Electrochemical Properties and Semiconductive Tendency in Acidic Chloride Solutions Under Dynamic Conditions

**Figure 8:**
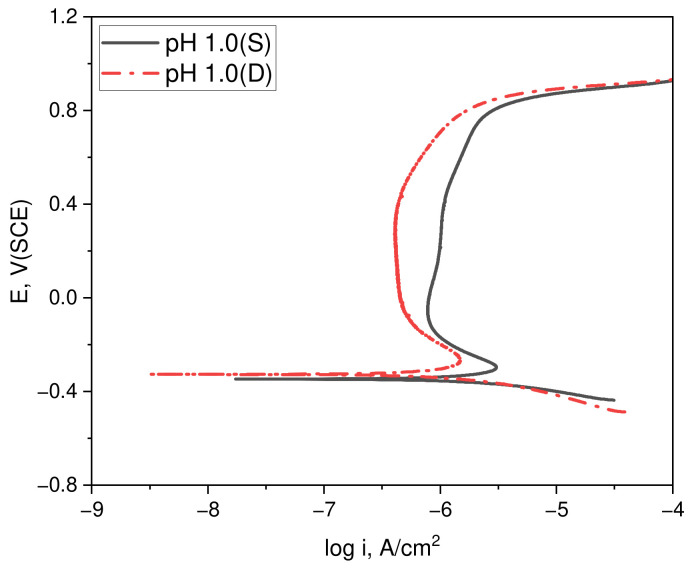
Effect of static and dynamic solution conditions on the polarization behavior of duplex stainless steel.

Figure 8 depicts the polarization results of duplex stainless steel in both static and dynamic acidic chloride solution conditions. In the static condition, the experiment was initiated immediately after solution preparation, while in the dynamic condition, an anodic polarization was carried out up to a current density that would not damage the specimen in a neutral chloride solution before injecting 0.1N HCl to transition to an acidic environment. The specimens were then stabilized for 10 min at a potential of −100 mV (SCE), within the passive film region, prior to the anodic polarization test. The polarization experiments in both the dynamic and static solutions revealed corrosion potentials (E_R_) of −0.35 V (SCE) and −0.33 V (SCE), corrosion current densities (i_R_) of 1.39 μA/cm^2^ and 0.52 μA/cm^2^, and passive current densities (i_P_) of 0.79 μA/cm^2^ and 0.55 μA/cm^2^ at a potential of −100 mV (SCE). The transpassive potential (E_tr_) was consistent at 0.82 V (SCE) under both conditions, suggesting similar polarization characteristics between the dynamic and static environments.

**Figure 9:**
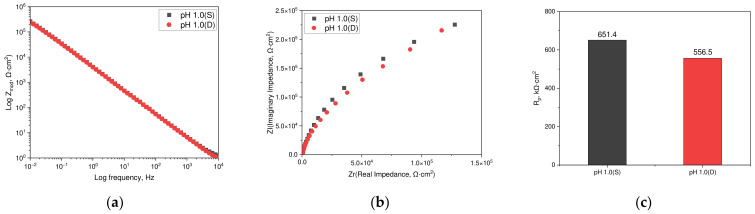
Effect of static and dynamic solution conditions on the AC impedance of duplex stainless steel formed at −100 mV (SCE): (**a**) Bode plot, (**b**) Nyquist plot, and (**c**) polarization resistance by CPE model.

Figure 9 illustrates the AC impedance results for the passive films on duplex stainless steel formed at a potential of −100 mV (SCE) for 1 h under static and dynamic solution conditions. The dynamic acidic chloride condition was conducted under the same parameters as the anodic polarization test, subsequent to the AC impedance test in neutral chloride solutions. Figure 9a displays the Bode plot and Figure 9b shows the Nyquist plot; both plots demonstrate that the results under static and dynamic conditions were remarkably similar. Figure 9c presents the calculated polarization resistance values from analyzing Figure 9a,b using the Randles model, with the measured polarization resistance at 651.4 kΩ·cm^2^ in the static environment and 556.5 kΩ·cm^2^ in the dynamic environment. The polarization resistance in dynamic environments was slightly lower but not significantly so.

**Figure 10:**
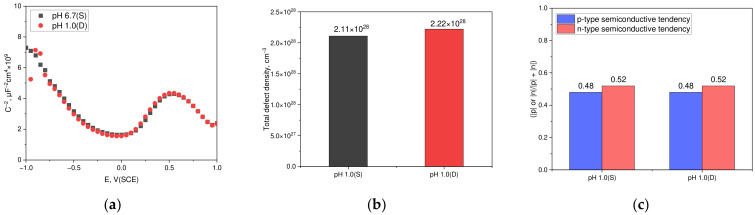
Effect of static and dynamic solution conditions on the Mott–Schottky characteristics of the passive film formed on duplex stainless steel at −100 mV (SCE): (**a**) Mott–Schottky plot, (**b**) pH vs. total defect density, and (**c**) pH vs. p-type or n-type semiconductive tendency.

Figure 10 depicts the impact on the Mott–Schottky properties for the passive films of duplex stainless steel formed at a potential of −100 mV (SCE) for 1 h under static and dynamic solution conditions. The Mott–Schottky test was conducted in a neutral chloride solution and proceeded under the same conditions as the anodic polarization test under dynamic acidic chloride conditions. Figure 10a illustrates the Mott–Schottky results for the passive films formed in both environments. The behavior exhibited almost identical characteristics in both environments, indicated by a negative slope showing p-type characteristics ranging from −0.85 V (SCE) to −0.4 V (SCE) and a positive slope depicting n-type characteristics from 0.2 V (SCE) to 0.4 V (SCE). The semiconductor properties, analyzed with the point defect model and bipolar model in static and dynamic conditions, are shown in Figure 10b,c. A comparison using these models reveals nearly consistent semiconductor properties in both environments.

An XPS analysis was conducted to examine the chemical states of the elements in the outer and inner layers of the passive films formed under dynamic and static conditions in acidic chloride solutions. This analysis was performed with the etching times set uniformly at 20 and 80 s, respectively, for both conditions.

**Figure 11:**
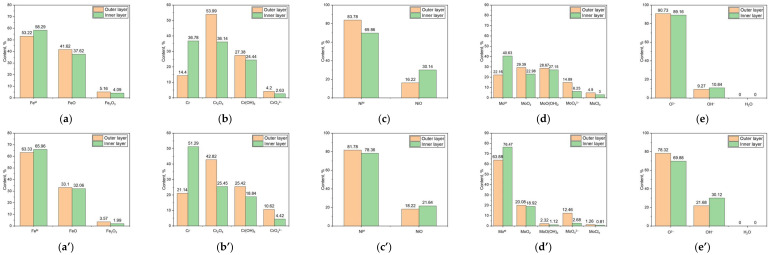
(**a**,**a’**) Fe 2p_3/2_, (**b**,**b’**) Cr 2p_3/2_, (**c**,**c’**) Ni 2p_3/2_, (**d**,**d’**) Mo (3d_5/2_ + 3d_3/2_), and (**e**,**e’**) O 1s elemental composition of outer and inner layers of passive films under (**a**–**e**) static condition and (**a’**–**e’**) dynamic condition.

Figure 11 shows the Fe 2p_3/2_, Cr 2p_3/2_, Ni 2p_3/2_, Mo (3p_5/2_ + 3p_3/2_), and O 1s elemental composition of the outer and inner layers of the passive films under static and dynamic conditions. Figure 11a,a’ illustrate the Fe 2p_3/2_ chemical states in the outer and inner layers of the passive films formed under both static and dynamic conditions in an acidic chloride environment. These chemical states manifested as Fe^M^, FeO, and Fe_2_O_3_, irrespective of the conditions. The concentrations of Fe^M^ increased toward the inner layers of the passive film under both conditions, while FeO and Fe_2_O_3_ were predominantly found in the outer layer under similar conditions. Figure 11b,b’ display the Cr 2p_3/2_ chemical states in both the outer and inner layers of the passive films formed under static and dynamic conditions in an acidic chloride setting. The Cr 2p_3/2_ chemical states included Cr^M^, Cr_2_O_3_, Cr(OH)_3_, and CrO_4_^2−^, maintaining consistency across various conditions. The Cr^M^ concentrations increased toward the inner layers of the passive film in both conditions, more so under the dynamic conditions. Conversely, Cr_2_O_3_, Cr(OH)_3_, and CrO_4_^2−^ were predominantly located in the outer layer under both conditions. Figure 11c,c’ depict the Ni 2p_3/2_ chemical states in the outer and inner layers of the passive films formed in an acidic chloride environment under static and dynamic conditions. The Ni 2p_3/2_ chemical states, consisting of Ni^M^ and NiO, remained consistent regardless of the conditions. Ni^M^ is primarily localized in the outer layer and NiO in the inner layer of the passive film under both conditions. Figure 11d,d’ reveal the Mo (3p_5/2_ + 3p_3/2_) chemical states in the outer and inner layers of the passive films formed under static and dynamic conditions in an acidic chloride environment. The Mo (3p_5/2_ + 3p_3/2_) chemical states included Mo^M^, MoO_2_, MoO(OH)_2_, MoO_4_^2−^, and MoCl_2_, independent of the conditions. MoO_2_, MoO(OH)_2_, MoO_4_^2−^, and MoCl_2_ were primarily found in the outer layer, while concentrations of Mo^M^ increased toward the inner layer under both conditions. Figure 11e,e’ illustrate the O 1s chemical states in both the outer and inner layers of the passive films formed under static and dynamic conditions in an acidic chloride environment. The chemical states of O 1s were consistently identified as O^2−^ and OH^−^, regardless of these conditions. O^2−^ was predominantly found in the outer layer under both static and dynamic conditions, while OH^−^ was more prevalent in the inner layer, independent of the conditions. H_2_O was not detected under either condition.

**Figure 12:**
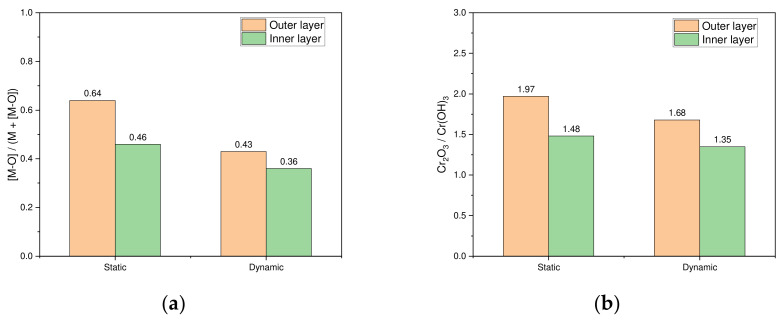
Comparison of [M-O]/(M + [M-O]) and Cr_2_O_3_/Cr(OH)_3_ ratios for outer and inner layers of passive films formed under static and dynamic conditions: (**a**) ratio of [M-O]/(M + [M-O]) and (**b**) ratio of Cr_2_O_3_/Cr(OH)_3_.

Figure 12 displays the [M-O]/(M + [M-O]) ratios and Cr_2_O_3_/Cr(OH)_3_ ratios for the outer and inner layers of the passive films formed under both static and dynamic conditions in an acidic chloride environment. Figure 12a presents the [M-O]/(M + [M-O]) ratio for each condition, showing a decreasing trend from the outer layer to the inner layer. Likewise, Figure 12b illustrates the Cr_2_O_3_/Cr(OH)_3_ ratios in each condition, which also decline from the outer layer to the inner layer. This suggests that the oxide distribution and ratios in passive films formed under static conditions are nearly consistent with those formed under dynamic conditions. These findings are consistent with the results reported in previous studies [11,12,13,14,28,30,37].

## 4. Conclusions

In this study, the electrochemical and semiconducting properties were analyzed under static and dynamic conditions in acidic (1N NaCl + 0.1N HCl, pH 1.0), neutral (1N NaCl, pH 6.7), and alkaline (1N NaCl + 0.1N NaOH, pH 13.2) chloride solutions. The investigation confirmed that the duplex stainless steels consistently exhibited similar passivation behavior (0.79 μA/cm^2^ > i_p_ > 0.2 μA/cm^2^ and 590 kΩ·cm^2^ < R_p_ < 651 kΩ·cm^2^). This led to the following conclusions.

(1) The semiconductive properties in neutral and alkaline chloride solutions demonstrated strong p-n-type semiconductive behavior and a weak semiconductive tendency balance, whereas in acidic chloride solutions they showed weak p-n-type semiconductive behavior and a strong semiconductive tendency balance. In other words, the reason why stainless steel, which exhibited stable passive behavior in neutral (pH 6.7) chloride solution, exhibited similar passive behavior in acidic (pH 1.0) chloride solution is due to the balance of the semiconductive tendencies of the passive film formed on the surface. Conversely, the outstanding passive behavior of passive films in neutral and alkaline solutions, despite their unbalanced semiconductive tendencies, is likely due to the low corrosivity of these solutions and the thickness of the passive films.
(2) The electrochemical and semiconductive properties evaluated under dynamic conditions from neutral to acidic chloride solutions were almost identical to those assessed under static conditions in the acidic chloride solution. These findings suggest that the passive film on the stainless steel surface adapts to environmental changes and can be spontaneously repassivated.

## Figures and Tables

**Table 1:** Chemical composition of duplex stainless steel.

Material	Chemical Compositions, wt.%									* PREN_30_
Duplex stainless steel	Cr	Mo	W	Si	Ni	Mn	C	N	Fe	46.1
	25.8	2.3	0.16	0.48	10.7	0.65	0.03	0.42	Bal	

* PREN_30_ (Pitting Resistance Equivalent Number) = %Cr + 3.3 (%Mo + 0.5%W) + 30%N.

**Table 2:** Electrochemical polarization factors of duplex stainless steel in different pH chloride solutions (S: static).

pH	E_R_, V(SCE)	i_R_, μA/cm^2^	E_tr_, V(SCE)	i_P_ at −100 mV(SCE), μA/cm^2^
1.0(S)	−0.35	+1.39	+0.83	+0.79
6.7(S)	−0.34	+0.02	+1.12	+0.35
13.2(S)	−0.56	+0.03	+0.49	+0.20

**Table 3:** Donor density, acceptor density, and total defect density of passive film on duplex stainless steel formed in different pH chloride solutions.

	pH 1.0 (1N NaCl + 0.1N HCl)	pH 6.7 (1N NaCl)	pH 13.2 (1N NaCl + 0.1N NaOH)
E_fb_ by P slope, V (SCE)	+0.14	−0.94	−1.12
E_fb_ by N slope, V (SCE)	−0.07	−0.41	−0.66
N_A_ (cm^−3^)	10.9 × 10^27^	1.89 × 10^27^	3.59 × 10^27^
N_D_ (cm^−3^)	10.2 × 10^27^	4.86 × 10^27^	2.24 × 10^27^
Total defect density (cm^−3^)	21.1 × 10^27^	6.76 × 10^27^	5.83 × 10^27^

## Data Availability

The original contributions presented in this study are included in the article. Further inquiries can be directed to the corresponding author.
